# Neuro-immune axis in atherosclerosis: mechanisms of regulation and therapeutic opportunities

**DOI:** 10.3389/fimmu.2025.1619338

**Published:** 2025-09-05

**Authors:** Yuxin Shang, Yuqing Pan, Lingling Xie, Yan Zhao, Wei Mao, Tingting Chen

**Affiliations:** ^1^ Zhejiang Key Laboratory of Integrative Chinese and Western Medicine for Diagnosis and Treatment of Circulatory Diseases, Zhejiang Hospital (Affiliated Zhejiang Hospital, Zhejiang University School of Medicine), Hangzhou, Zhejiang, China; ^2^ Zhejiang Engineering Research Center for Precise Diagnosis and Innovative Traditional Chinese Medicine for Cardiovascular Diseases, Zhejiang Hospital (Affiliated Zhejiang Hospital, Zhejiang University School of Medicine), Hangzhou, Zhejiang, China; ^3^ The First School of Clinical Medicine, Zhejiang Chinese Medical University, Hangzhou, Zhejiang, China; ^4^ Cardiovascular Department, Zhejiang Hospital (Affiliated Zhejiang Hospital, Zhejiang University School of Medicine), Hangzhou, Zhejiang, China

**Keywords:** atherosclerosis, immune, nervous system, mechanism, therapy

## Abstract

Atherosclerosis, the leading cause of cardiovascular morbidity and mortality worldwide, is now firmly established as a chronic immune-mediated disorder rather than a purely lipid-storage disease. Accumulating evidence has uncovered a previously underappreciated dimension of atherogenesis: the dynamic and bidirectional crosstalk between the nervous and immune systems. This neuroimmune axis, involving intricate communication between autonomic neural circuits and vascular immune cells, plays a central role in regulating arterial inflammation and plaque development. In particular, neuroimmune cardiovascular interfaces (NICIs)—specialized anatomical and functional hubs—have emerged as key sites for signal integration. Here, we review recent mechanistic insights into how sympathetic and parasympathetic pathways influence immune responses in atherosclerotic vessels and hematopoietic organs. We focus on the roles of neuromodulators such as pituitary adenylate cyclase-activating polypeptide (PACAP), calcitonin gene-related peptide (CGRP), neuropeptide Y (NPY), and galanin in shaping myeloid cell behavior, vascular tone, and endothelial activation. Additionally, we examine translational advances in neuromodulatory interventions—ranging from vagus nerve stimulation (VNS) to selective α7 nicotinic acetylcholine receptor (α7nAChR) agonists—that target these pathways to mitigate vascular inflammation in experimental models. These findings suggest that spatially resolved and temporally dynamic neuroimmune interactions constitute a critical layer of regulation in atherogenesis, offering a compelling framework for novel anti-inflammatory therapies beyond traditional lipid-lowering strategies.

## 1 Introduction

Atherosclerosis (AS) is a chronic inflammatory disease of the arterial wall and remains the leading cause of cardiovascular morbidity and mortality worldwide (1, 2). The disease originates from subendothelial accumulation of apolipoprotein B-containing lipoproteins (e.g. LDL), which triggers innate and adaptive immune responses leading to plaque formation (3). Despite advances in lipid-lowering and anti-inflammatory therapies, a significant proportion of patients continue to experience recurrent cardiovascular events (4, 5). This highlights an urgent need for novel therapeutic strategies that address not only lipid accumulation but also the underlying immune and inflammatory mechanisms driving disease progression.

Emerging evidence suggests that AS is not merely a lipid-driven disorder but also a chronic inflammatory disease shaped by neuroimmune dysregulation (6, 7). Among the neural regulators, the autonomic nervous system (ANS), comprising sympathetic (SNS) and parasympathetic (PNS) branches, plays a pivotal role in vascular inflammation. Chronic SNS activation enhances leukocyte recruitment and proinflammatory cytokine release via β-adrenergic signaling (8–10). On the other hand, PNS activity, particularly through vagus nerve, mediated cholinergic anti-inflammatory pathways, reduces inflammation by directly modulating macrophages, as shown in recent work indicating direct β_2_-adrenergic receptor (β_2_-AR) signaling to splenic myeloid cells, independent of a T-cell relay (11). This finding contrasts with the earlier paradigm proposed by Rosas-Ballina et al. (12), relaying vagal input to α7nAChR on macrophages. Notably, ChAT^+^ T cells have also been observed in ATLOs adjacent to adrenergic varicosities (7), suggesting that both mechanisms may operate in atherosclerosis but in context-dependent manners, direct β_2_-AR signaling may predominate in diffuse inflammatory milieus, whereas a ChAT^+^ T-cell relay could be engaged in structured adventitial lymphoid aggregates. Reconciling these pathways will require spatially resolved functional studies in vascular neuroimmune niches. Taken together, these contrasting effects, mediated by distinct neurotransmitters such as norepinephrine and acetylcholine, highlight the possible neuroimmune crosstalk relevant to AS pathophysiology (13, 14), rather than a uniformly established mechanism.

Specialized neuroimmune cardiovascular interfaces (NICIs) in the arterial adventitia enable direct communication between sensory nerves and immune aggregates, dynamically modulating local inflammation (7). Dysregulation of the neuro-immune axis, such as elevated corticotropin-releasing hormone (CRH) and reduced vagal activity, as measured by decreased heart rate variability and diminished efferent cholinergic signaling, which reflects reduced parasympathetic signaling through the vagus nerve, can exacerbate vascular inflammation and disrupt cardiovascular homeostasis (15, 16).

Therapeutically, targeting the neuroimmune axis has shown promise in preclinical models. For example, bioelectronic vagus nerve stimulation attenuates systemic inflammation via activation of the cholinergic anti-inflammatory pathway (17). Optogenetic approaches offer promising precision control of sympathetic circuits in neural regulation, but their direct therapeutic role in atherosclerosis remains to be investigated. On the other hand, clinical translation of neuromodulatory approaches remains limited by the difficulty of selectively targeting specific neural circuits without off-target effects or systemic interference. Emerging technologies, such as single-cell transcriptomics and spatial mapping, may help identify discrete neural-immune modules, offering new avenues for precise therapeutic targeting (18, 19).

This review synthesizes recent advances in understanding neuroimmune regulation in AS, with a particular focus on adventitial interfaces and macrophage plasticity. We further evaluate the therapeutic potential of both neuromodulatory and pharmacological strategies, highlight current limitations, and discuss future directions for precise, integrative interventions that bridge neural and immune modulation in vascular disease.

## 2 Immune dysregulation in atherosclerosis

**Figure 1:**
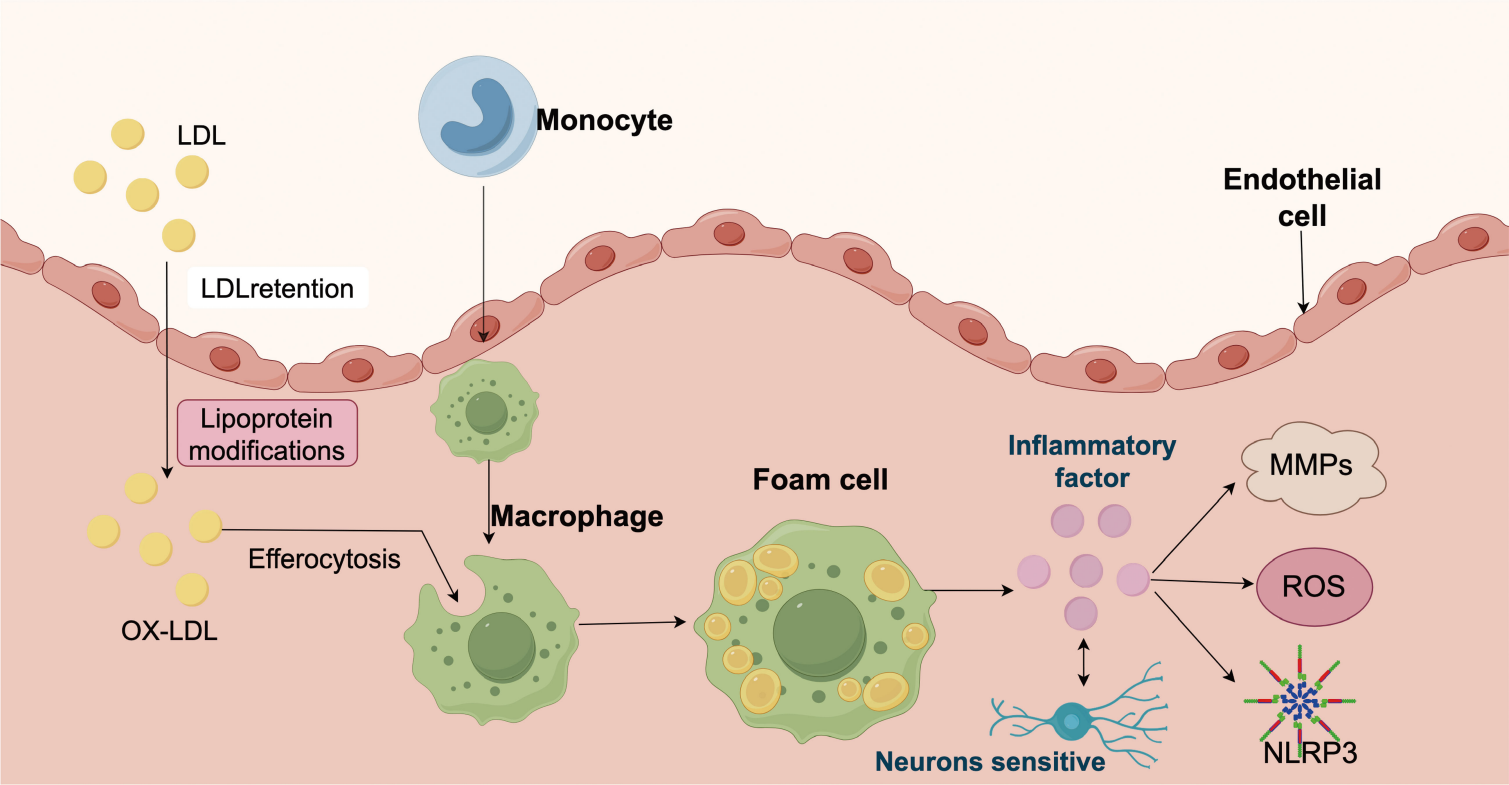
Schematic representation of the pathological mechanisms involved in atherosclerosis. Low-density lipoprotein (LDL) particles infiltrate the arterial intima, where they become retained and undergo various modifications, leading to the formation of oxidized LDL (ox-LDL). These modified lipoproteins trigger recruitment of circulating monocytes into the subendothelial space, where they differentiate into macrophages and engulf ox-LDL through efferocytosis. Lipid-laden macrophages transform into foam cells, a hallmark of early atherosclerotic lesions. Foam cells release inflammatory factors, which can activate endothelial cells, induce matrix metalloproteinases (MMPs), and stimulate the production of reactive oxygen species (ROS). These inflammatory mediators further amplify vascular inflammation, enhance oxidative stress, and activate the NLRP3 inflammasome. Activation of NLRP3 leads to further cytokine release, contributing to chronic inflammation and tissue remodeling. Sensory neurons in the vascular wall may also respond to inflammatory cues, suggesting a potential role in neuroimmune interactions during atherosclerosis progression.

AS begins with endothelial dysfunction, triggered by cardiovascular risk factors such as hyperlipidemia, hypertension, and oxidative stress (20). Dysfunctional endothelium promotes the retention and oxidation of low-density lipoprotein cholesterol (LDL-C) in the subendothelial space (21, 22). Oxidized LDL (oxLDL) acts as a damage-associated molecular pattern (DAMP), activating endothelial cells and inducing the expression of adhesion molecules, such as vascular cell adhesion molecule-1 (VCAM-1) and intercellular adhesion molecule-1 (ICAM-1) (23, 24). These molecules mediate the recruitment of circulating monocytes and lymphocytes, facilitating the recruitment of monocytes and lymphocytes (25, 26) (**Figure 1**).

Early innate immune activation, dominated by monocyte-derived macrophages, initiates lipid clearance but paradoxically sustains inflammation through cytokine secretion (27). Monocytes differentiate into macrophages upon entering the intima, where they engulf oxidized LDL to form foam cells, which is a hallmark of early fatty streaks (28, 29). Foam cells secrete pro-inflammatory cytokines, including tumor necrosis factor-alpha (TNF-α) and interleukin-1 beta (IL-1β), which amplifies local inflammation and promotes further leukocyte recruitment (30). Activated macrophages further release reactive oxygen species (ROS) and matrix metalloproteinases (MMPs), driving oxidative stress and extracellular matrix degradation, thereby weakening the fibrous cap and increase the risk of plaque rupture (31, 32).

Transition from fatty streaks to complex plaques involves macrophage phenotypic plasticity: pro-inflammatory M1 subsets exacerbate necrotic core formation via NOD-like receptor thermal protein domain associated protein 3 (NLRP3) inflammasome activation (33). In contrast, alternatively activated M2 macrophages secrete TGF-β and promote tissue repair and fibrosis (34). Meanwhile, Adaptive immune responses are also engaged. Th1 and Th17 cells recognize modified self-antigens such as apoB-100, releasing INF-γ and IL-17 to perpetuate inflammation and impair vascular integrity (35).

## 3 Immunopathological crosstalk

The progression of AS is fundamentally orchestrated by a dynamic crosstalk between innate and adaptive immune cells within the arterial wall. Endothelial dysfunction triggered by oxLDL and hemodynamic stress initiates a cascade of immune recruitment and activation that dictates plaque evolution.

### 3.1 Innate immune orchestrators

**Figure 2:**
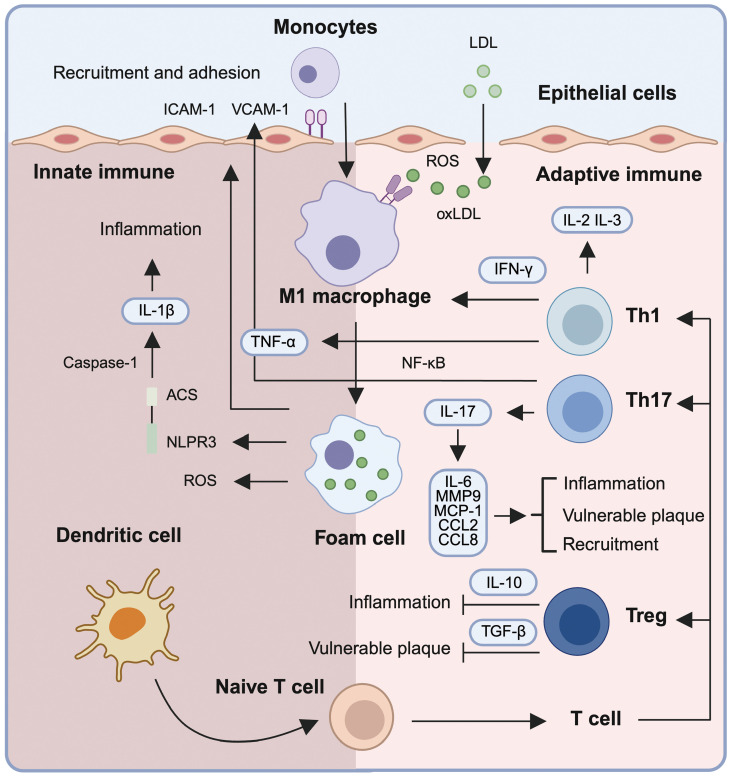
Immune mechanisms driving inflammation and plaque vulnerability in atherosclerosis. Low-density lipoprotein (LDL) infiltrates the subendothelial space and undergoes oxidative modification by reactive oxygen species (ROS), forming oxidized LDL (oxLDL). oxLDL triggers the upregulation of adhesion molecules (ICAM-1 and VCAM-1) on endothelial cells, facilitating monocyte recruitment and trans-endothelial migration. Within the intima, monocytes differentiate into M1 macrophages that internalize oxLDL, forming lipid-laden foam cells. These macrophages amplify inflammation via secretion of pro-inflammatory cytokines such as TNF-α and IL-1β, the latter being activated through the NLRP3 inflammasome. Foam cells and activated macrophages release mediators (e.g., IL-6, MCP-1, CCL2, CCL8, MMP9) that contribute to immune cell recruitment, plaque instability, and chronic vascular inflammation. Dendritic cells present antigens to naïve T cells, initiating adaptive immune responses. T helper (Th) subsets including Th1 and Th17 secrete IFN-γ and IL-17, respectively, further propagating inflammation and destabilizing plaques. In contrast, regulatory T cells (Tregs) produce IL-10 and TGF-β, exerting anti-inflammatory effects and promoting immune homeostasis. This coordinated interplay between innate and adaptive immune cells drives atherosclerotic progression and highlights potential targets for immunomodulatory therapy.

Monocytes and macrophages are the dominant innate effectors in early atherogenesis (**Figure 2**). oxLDL-induced endothelial activation upregulates VCAM-1 and ICAM-1, facilitating monocyte adhesion and transendothelial transmigration (36). Intimal monocytes differentiate into macrophages that internalize oxLDL via scavenger receptors (e.g., CD36, LOX-1), forming lipid-laden foam cells, which is a process amplified by pro-inflammatory cytokines such as IL-1β and TNF-α (37, 38). This process amplifies local inflammation and recruiting additional immune cells and destabilizes the plaque microenvironment. In parallel, macrophages-derived ROS, contributes to matrix degradation and promote necrotic core formation (39).

Dendritic cells (DCs) are another critical innate immune cell type, functioning as antigen-presenting cells (APCs) that process and present oxLDL-derived antigens to T cells (40, 41). Both conventional dendritic cells (cDCs) and plasmacytoid dendritic cells (pDCs) are involved in AS by promoting antigen-specific T cell activation, pro-inflammatory responses, and loss of tolerance (42, 43). The maturation of plaque DCs, driven by inflammatory stimuli and necrotic signals, contributes to atherogenesis through enhanced T cell activation and reduced anti-inflammatory signaling (**Figure 2**) (44, 45).

### 3.2 Adaptive immune modulators

CD4+ T cell subsets play divergent roles in atherogenesis: Th1 cells secrete IFN-γ to sustain macrophage activation, whereas Th17-derived IL-17 promotes endothelial dysfunction (**Figure 2**) (46, 47). Regulatory T cells (Tregs) counterbalance inflammation via the production of interleukin-10 (IL-10) and transforming growth factor-beta (TGF-β), yet their stability and function are impaired in the inflammatory plaque milieu due to oxLDL-induced endoplasmic reticulum stress and IL-6-mediated suppression of FoxP3 expression (48, 49).

B cells also contribute to plaque dynamics in a subset-specific manner. B2 cells exacerbate atherosclerosis through the production of IgG antibodies that that engage Fcγ receptors on macrophages, enhancing macrophage lipid uptake and inflammatory activation, while B1 cells produce IgM antibodies that facilitate apoptotic cell clearance and neutralize modified lipids, conferring atheroprotective effects (50, 51).

## 4 Neuroimmune communication in physiological states

**Figure 3:**
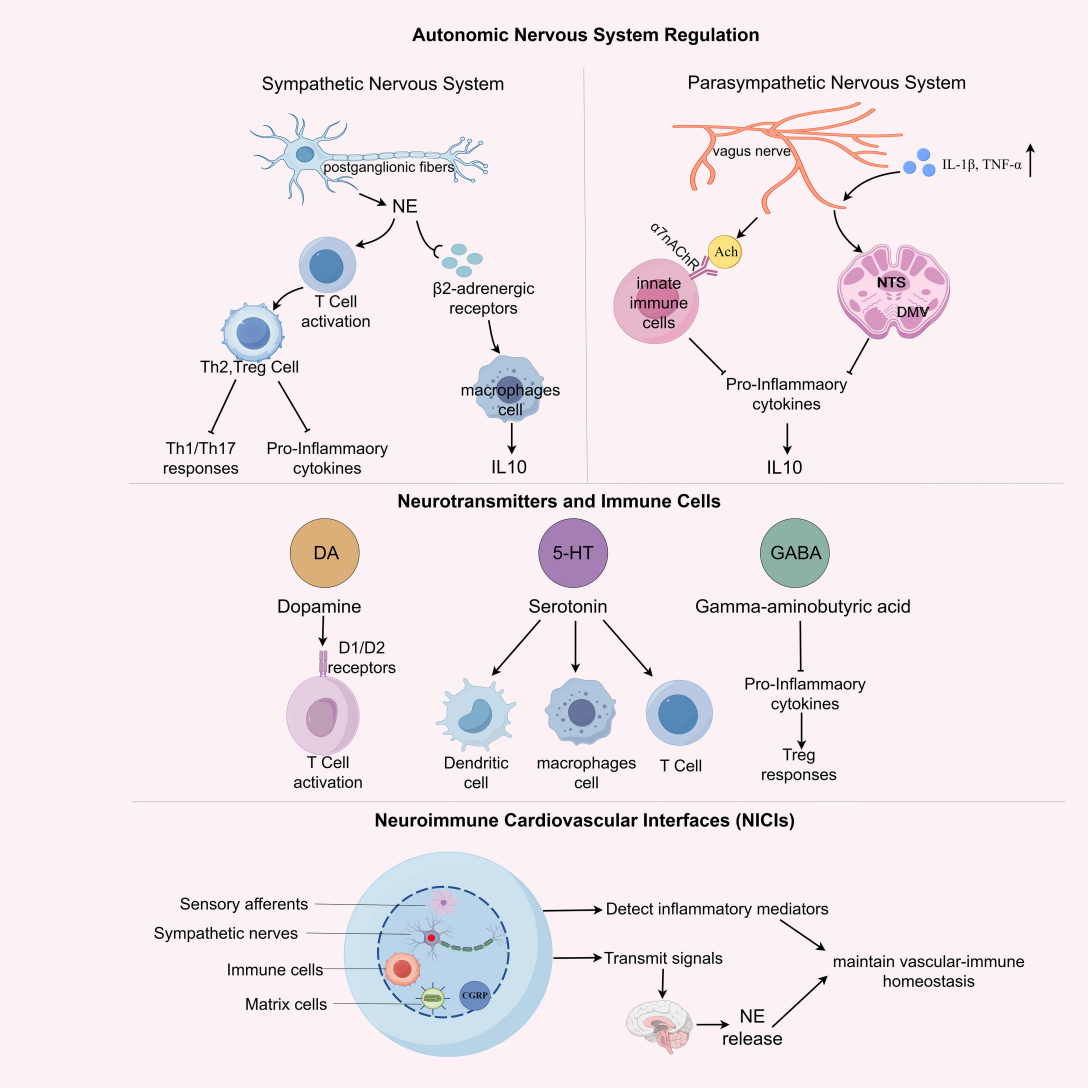
Neuroimmune regulation in physiological homeostasis. This schematic illustrates the coordinated regulation of immune function by the autonomic nervous system (ANS), neuromodulatory transmitters, and neuroimmune cardiovascular interfaces (NICIs) under physiological conditions. The sympathetic nervous system (SNS) exerts pro-inflammatory effects via norepinephrine (NE) and β2-adrenergic receptor (β2-AR) signaling on T cells and macrophages, favoring Th1/Th17 polarization and production of pro-inflammatory cytokines (e.g., TNF-α, IL-1β). In contrast, the parasympathetic nervous system (PNS), primarily via the vagus nerve, engages anti-inflammatory pathways through acetylcholine release and α7 nicotinic acetylcholine receptor (α7nAChR) activation on immune cells, promoting Th2/Treg responses and IL-10 secretion. Central neuromodulators such as dopamine, serotonin, and γ-aminobutyric acid (GABA) further influence immune cell activation, shaping T cell phenotypes and dendritic cell function. At the structural interface between nerves and immune cells, NICIs enable bidirectional crosstalk: sensory afferents detect local inflammatory cues, while efferent autonomic fibers modulate immune activity via neurotransmitter release into the perivascular niche. Together, these multi-layered neural mechanisms maintain vascular-immune homeostasis and prepare the groundwork for neuroimmune dysregulation in disease.

Bidirectional communication between the nervous and immune systems is essential for maintaining homeostasis. Though a network of neurotransmitters, neuropeptides, and signaling pathways, the ANS, sensory fibers, and central neural circuits dynamically regulate immune cell development, activation, and trafficking. This section outlines key physiological mechanisms by which neural inputs influence immune function (**Figure 3**).

### 4.1 Autonomic neuro-immune regulation

The SNS and PNS represent the major autonomic branches regulating immune homeostasis.

The SNS modulates immune responses primarily through the release of norepinephrine (NE) from postganglionic fibers innervating lymphoid organs, including bone marrow, spleen, and lymph nodes. NE binds to β2-adrenergic receptors (β2-ARs) expressed on various immune cells (52). In macrophages, β2-AR activation suppresses pro-inflammatory cytokines and promotes anti-inflammatory IL-10 production, shifting them toward an M2-like phenotype (53). In CD4^+^ T cells, NE signaling enhances differentiation toward Th2 and Treg subsets, while dampening Th1/Th17 responses and inflammatory cytokine release (54).

Conversely, the PNS, primarily via the vagus nerve, exerts anti-inflammatory control through the cholinergic anti-inflammatory pathway. Acetylcholine (ACh), the primary neurotransmitter of the vagus nerve, binds to α7 nicotinic acetylcholine receptors (α7nAChR) on innate immune cells, particularly macrophages and dendritic cells, or potentially at upstream autonomic ganglia such as the celiac ganglion, as suggested by recent work (11). While α7nAChR signaling can downregulate TNF-α and IL-1β and enhance IL-10 production (55), the precise anatomical locus of this modulation remains incompletely defined and may vary across inflammatory contexts.

In parallel, vagal afferent fibers can detect peripheral inflammatory mediators, such as IL-1β and TNF-α, and relay these signals to the nucleus tractus solitarius (NTS) and dorsal motor nucleus of the vagus (DMV), thereby initiating a reflexive efferent anti-inflammatory output that constrains systemic immune activation (56).

Together, these autonomic circuits calibrate immune surveillance and tolerance in steady-state conditions, allowing the body to rapidly respond to threats while preventing excessive inflammation.

### 4.2 Neurotransmitters and immune cells

Beyond NE and ACh, a range of classical neurotransmitters and neuropeptides act as immune modulators.

Dopamine (DA) modulates both innate and adaptive immune responses. D1-like receptor signaling has been linked to enhanced T cell activation and pro-inflammatory cytokine release, whereas D2-like receptor activity promotes the generation and function of Tregs, thus maintaining immune homeostasis (57, 58). In APCs, DA regulates IL-12 and IL-23 expression in a receptor-dependent manner: activation of D1-like receptors, particularly D5R, enhances IL-12 and IL-23 production (via STAT3 inhibition), whereas absence of D5R markedly reduces these cytokines, illustrating how receptor subtype and local concentration shape APC-driven adaptive immunity (59).

Serotonin (5-HT), derived from both platelets and enterochromaffin cells, regulates dendritic cell maturation, macrophage chemotaxis, and T cell polarization. It exerts its effects through multiple 5-HT receptor subtypes, which are differentially expressed across immune cell types (60, 61). Serotonin signaling is also implicated in modulating vascular permeability and platelet aggregation, thereby linking neuroimmune signaling with early inflammatory responses (62, 63).

Gamma-aminobutyric acid (GABA), the principal inhibitory neurotransmitter in the CNS, also modulates immune responses. GABA A and GABA B receptors are expressed on T cells and macrophages (64). GABAergic signaling suppresses pro-inflammatory cytokine production, inhibits antigen presentation, and promotes regulatory T cell responses. GABA analogs and receptor agonists are currently under investigation for their potential to modulate neuroinflammation and peripheral immune responses in chronic inflammatory diseases (64–66). Together, these neurotransmitters form a regulatory axis that enables context-dependent modulation of immune function across peripheral and central compartments.

### 4.3 Neuroimmune cardiovascular interfaces as structural hubs

NICIs have recently been identified as specialized sites in the adventitia of large arteries, including the aorta and carotids. These interfaces consist of sensory afferents, sympathetic nerve fibers, resident immune cell clusters (e.g., macrophages, DCs), and stromal cells. NICIs function as neuroimmune synapse-like structures that permit real-time communication between vascular inflammation and central neural circuits (7).

In healthy vessels, NICIs contribute to homeostasis by regulating immune tone and facilitating vessel wall monitoring. Afferent nociceptive neurons detect inflammatory mediators (e.g., IL-1β, CCL2) and relay signals to the brainstem, particularly the nucleus tractus solitarius (NTS), thereby initiating autonomic feedback loops. Sympathetic efferents project back to the same vascular sites, releasing NE locally to modulate immune cell activation, antigen presentation, and cytokine production (7).

In addition to neurotransmitters, NICIs also house neuropeptides such as calcitonin gene-related peptide (CGRP), which has been shown to influence endothelial barrier function and shift macrophage phenotype (67, 68). In healthy arteries, functionally relevant NICIs are absent and arise *de novo* during disease onset, but their structural and functional plasticity makes them susceptible to extensive remodeling during pathological states such as atherosclerosis, hypertension, and autoimmune vasculitis. This potential for pathological rewiring underscores their importance as early sentinels of immune dysregulation.

## 5 Neuroimmune dysregulation and atherosclerosis pathophysiology

**Figure 4:**
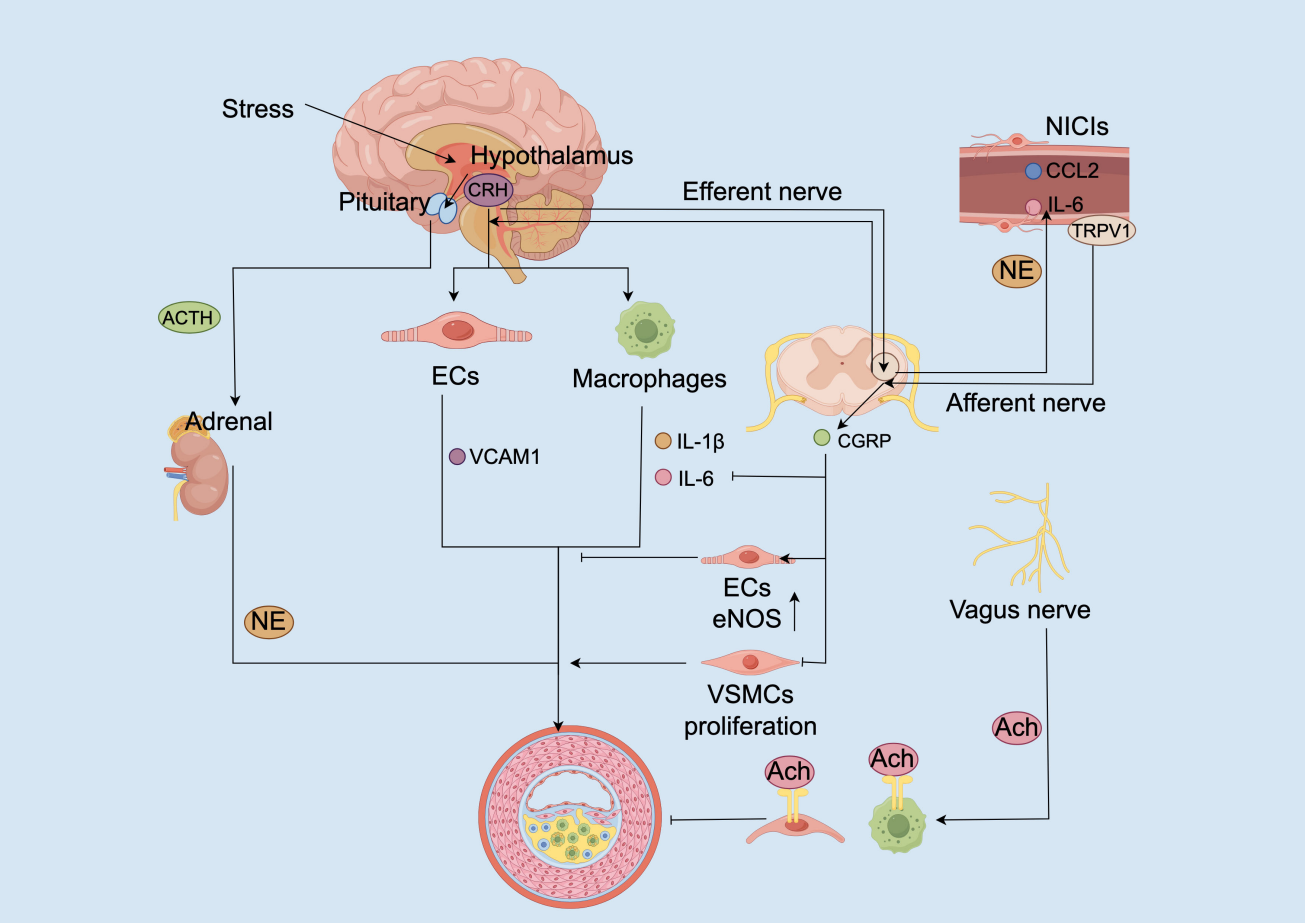
Neuroimmune circuit dysregulation promotes vascular inflammation and atherogenesis. Chronic stress activates the hypothalamic–pituitary–adrenal (HPA) axis, leading to corticotropin-releasing hormone (CRH) secretion from the hypothalamus. CRH acts directly on endothelial cells (ECs) and macrophages to upregulate vascular cell adhesion molecule-1 (VCAM1) and stimulate pro-inflammatory cytokine release (e.g., IL-1β, IL-6). Concurrently, CRH stimulates adrenocorticotropic hormone (ACTH) release from the pituitary, promoting adrenal norepinephrine (NE) production. NE further exacerbates immune activation through β-adrenergic signaling. In peripheral neuroimmune cardiovascular interfaces (NICIs), stress-induced norepinephrine and inflammatory cues (e.g., IL-6, CCL2) sensitize TRPV1 channels on afferent sensory terminals, leading to CGRP release. CGRP promotes vasodilation and inhibits cytokine production in macrophages and dendritic cells. Efferent vagal signaling through acetylcholine (Ach) activates α7 nicotinic acetylcholine receptors (α7nAChRs) on macrophages and ECs, suppressing inflammation and enhancing eNOS expression. Imbalances in these neuroimmune loops, characterized by sympathetic hyperactivity, reduced vagal activity, and sustained TRPV1 sensitization, converge to promote EC dysfunction, vascular smooth muscle cell (VSMC) proliferation, and plaque progression.

In atherosclerosis, the delicate balance between neural and immune regulation is disrupted, fueling chronic vascular inflammation and plaque progression (**Figure 4**).

### 5.1 Dysregulation of autonomic control in atherosclerosis

Chronic psychosocial stress is increasingly recognized as a non-traditional but powerful accelerator of atherosclerosis through coordinated dysregulation of both the hypothalamic–pituitary–adrenal (HPA) axis and the autonomic nervous system. Activation of the HPA axis leads to elevated levels of CRH, which not only orchestrates systemic glucocorticoid responses but also exerts direct proinflammatory effects at the vascular level—promoting endothelial hyperpermeability and enhancing pro-inflammatory cytokine release from macrophages (69). Study has demonstrated that CRH directly promotes atherosclerosis progression by inducing endothelial inflammation. In LDLR^⁻/⁻^ mice, exogenous CRH administration significantly exacerbated plaque development, accompanied by increased VCAM-1 expression and activation of the NF-κB signaling pathway, which together facilitate leukocyte recruitment. These pro-atherogenic effects were receptor-dependent and effectively suppressed by pharmacological inhibition of CRH receptor 1 (CRHR1). Importantly, this CRH–CRHR1 signaling pathway acts independently of systemic lipid metabolism, indicating a local vascular mechanism contributing to atherogenesis beyond classical metabolic factors (70).

Concomitantly, stress reduces parasympathetic tone, impairing the cholinergic anti-inflammatory reflex that is essential for limiting vascular inflammation. In this pathway, acetylcholine—originating from neuronal or potentially non-neuronal sources—binds to α7nAChRs on macrophages and endothelial cells, a receptor interaction supported by evidence from *in vivo* and *in vitro* studies demonstrating suppression of pro-inflammatory cytokine production (11, 55). However, this regulatory pathway is often compromised in chronic inflammatory states. In ApoE^⁻/⁻^ mice, functional impairment of baroreflex control through sino-aortic denervation significantly exacerbates plaque burden, which is partially rescued by administration of selective α7nAChR agonists. This supports a critical role for vagal integrity in restraining local immune activation and endothelial dysfunction during atherogenesis (71).

Conversely, SNS overactivity enhances leukocyte recruitment and proinflammatory polarization via local NE release, primarily through β1-adrenergic receptor (β1-AR) and α-AR activation. Chronic SNS activation promotes myelopoiesis via β1-AR, increasing circulating inflammatory monocytes that infiltrate atherosclerotic plaques, effects reversed by non-selective β-adrenergic blockade (targeting both β1- and β2-AR) (72). NE also modulates neutrophil function and polarization, skewing them toward an immunosuppressive N2 phenotype while reducing their proinflammatory activity, although the precise implications of this polarization in atherosclerosis remain context-dependent (73).

Importantly, these autonomic imbalances do not occur in isolation but contribute to a pathophysiological feed−forward loop. CRH can directly enhance macrophage pro−inflammatory cytokine production, as shown by augmented TNF−α, IL−1β and IL−6 responses in LPS−stimulated macrophages (74). Meanwhile, catecholamines released from sympathetic nerve activation exhibit rhythmic, tissue-specific modulation of leukocyte adhesion to the vascular endothelium, promoting localized vascular inflammation with circadian variations (75). Notably, chronic inflammation is associated with increased sympathetic innervation density in the perivascular adventitia—suggesting neural remodeling reinforces immune activation (7). Collectively, these findings support a circuit in which autonomic imbalance, excessive sympathetic tone coupled with impaired parasympathetic anti-inflammatory signaling, facilitates vascular inflammation and plaque progression.

### 5.2 Neuromodulators in atherosclerosis

A growing body of experimental evidence supports the pivotal role of neuropeptides in orchestrating local immune responses in atherosclerosis. Among these, pituitary adenylate cyclase-activating polypeptide (PACAP), particularly its predominant isoform PACAP-38, has emerged as a key anti-inflammatory neuromodulator. In PACAP-deficient ApoE^⁻/⁻^ mice (PACAP^⁻/⁻^:ApoE^⁻/⁻^), plaques exhibit significantly increased area, macrophage infiltration, and foam cell formation, accompanied by elevated TNF-α expression, indicating that endogenous PACAP constrains proinflammatory macrophage activation (76). Activation of its high-affinity receptor PAC1 using the selective agonist Maxadilan attenuates plaque burden by over 40%, reduces apoptotic cell accumulation, and suppresses IL-1β and TNF-α in LDLR^⁻/⁻^ mice, without altering circulating lipids, suggesting local immunomodulatory effects (76). These findings underscore PACAP–PAC1 signaling as a vascular anti-inflammatory pathway with translational potential (77).

Beyond PACAP, other neuromodulators are increasingly recognized as crucial players in vascular immune regulation. Neuropeptide Y (NPY), highly expressed in sympathetic nerves, promotes vascular smooth muscle cell (VSMC) proliferation and M1 macrophage polarization (78). In a rat model of carotid balloon injury, slow-release NPY delivery induced occlusive atherosclerotic-like lesions containing lipid cores, macrophage infiltrates, thrombi, and neovessels, despite the absence of metabolic abnormalities or atherogenic diet. These effects were significantly attenuated by selective blockade of the Y1 receptor, with partial efficacy by Y5 receptor inhibition, implicating Y1R-mediated signaling as a key driver of NPY-induced vascular pathology (79).

Substance P (SP), via NK1 receptors, exerts dual effects depending on disease phase. In early atherogenesis, SP enhances monocyte recruitment and proinflammatory cytokine release, whereas in chronic stages it may promote tissue repair by stimulating Treg responses (80). Similarly, Secretoneurin is a pleiotropic peptide that promotes endothelial regeneration and angiogenesis but may also facilitate VSMC migration and contribute to fibrous cap destabilization (81).

Other modulators such as brain-derived neurotrophic factor (BDNF) and Galanin add further complexity. BDNF, traditionally known for its role in neuronal plasticity, is also expressed in human atherosclerotic plaques, particularly within smooth muscle cells, macrophages, and fibroblasts. Clinical imaging and immunohistochemistry studies have shown that elevated plasma BDNF levels correlate with increased macrophage infiltration in coronary plaques, suggesting a potential link to plaque vulnerability. Additionally, *in vitro* studies indicate that BDNF can enhance NAD(P)H oxidase activity in vascular cells, implicating a role in promoting oxidative stress within the vascular (82). Galanin is a neuropeptide that modulates autonomic tone and metabolic function and exhibits context-dependent immunoregulatory properties. In human monocytes and macrophages, galanin induces either pro-inflammatory (e.g., TNF-α, IL-1β, CXCL8) or anti-inflammatory (e.g., IL-10) cytokine expression depending on the activation state, demonstrating its bidirectional immune effects (83). Moreover, signaling through the GAL3 receptor has been shown to enhance the responsiveness of innate immune cells to inflammatory cues in specific inflammatory contexts, further supporting a potential pro-inflammatory role of galanin in chronic vascular inflammation (84).

Together, these findings suggest that atherosclerosis is modulated by a dynamic array of neuropeptides with context- and cell-specific effects. PACAP plays dominant anti-inflammatory roles, whereas NPY and SP may exacerbate inflammation depending on timing and receptor signaling. Understanding the spatial and temporal orchestration of these neuromodulators—and their interactions with neuroimmune interfaces such as NICIs, offers new opportunities for precision therapy.

### 5.3 Neuroimmune cardiovascular interfaces in atherosclerosis

Emerging evidence reveals that atherosclerotic inflammation induces profound structural and functional remodeling of the vascular adventitia, giving rise to specialized NICIs. In advanced lesions of both ApoE^⁻/⁻^ mice and human coronary arteries, high-resolution imaging has demonstrated dense networks of sympathetic and sensory nerve fibers interwoven with clusters of macrophages, T and B lymphocytes, and smooth muscle cells in the adventitial layer, forming integrated hubs of bidirectional artery–brain communication (7, 75).

NICIs play dual and dynamic roles in coordinating neural and immune signals. First, afferent sensory fibers expressing TRPV1 and CGRP detect inflammatory cues and transmit them via dorsal root and nodose ganglia to autonomic centers in the brainstem, parabrachial nucleus, and hypothalamus (7). Second, efferent sympathetic pathways descend from these regions to the vascular adventitia through spinal and celiac ganglia, where they release NE, modulating leukocyte behavior and endothelial activation (75). Ablation of the celiac ganglion in aged atherosclerotic mice disrupted NICI integrity, attenuated local immune activation, and reduced plaque progression, indicating that this peripheral neural circuit contributes to disease progression at late stages (7).

Within NICIs, the TRPV1–CGRP axis functions as a key sensory immunomodulatory pathway. In the context of atherosclerosis, inflammatory cues such as LPS/TLR4 signaling, local acidosis, and endogenous lipid mediators, including capsaisin, anandamide (AEA) and N-arachidonoyl dopamine (NADA), sensitize TRPV1 channels expressed on perivascular sensory afferents (85–87). Upon activation, TRPV1 induces the release of CGRP, a vasodilatory neuropeptide that suppresses pro-inflammatory cytokine production, notably TNF-α in macrophages and IL-12 in dendritic cells (88, 89). Although specific suppression of CXCL2 has not been directly demonstrated, these findings collectively support a broader anti-inflammatory role for CGRP in myeloid cell regulation. Moreover, CGRP enhances endothelial nitric oxide synthase (eNOS) activity and inhibits NF-κB–dependent chemokine expression, such as CCL2 and CXCL8, thus limiting monocyte adhesion and transmigration (90). Additionally, CGRP restrains smooth muscle cell proliferation and reducing neointimal hyperplasia (91). Of note, prolonged TRPV1 activation may induce receptor desensitization through calcium-dependent dephosphorylation mechanisms, potentially weakening its neuroprotective output in chronic inflammation settings (92).

Crucially, NICIs structurally integrate with adventitial tertiary lymphoid organs (ATLOs), which arise in response to chronic vascular inflammation and whose development depends on *de novo* innervation from sympathetic and sensory fibers, as demonstrated in recent experimental models (7, 93). Such nerve ingrowth not only provides structural linkage but also enables bidirectional communication between vascular sensory/autonomic circuits and ATLO-resident immune cells, potentially shaping local antigen presentation, cytokine milieus, and lymphoid organization during atherogenesis. In aged ApoE^⁻/⁻^ mice, ATLOs emerge as organized immune aggregates within the adventitia, comprising B-cell follicles, germinal centers, and high endothelial venules (93). study reported extensive nerve fiber sprouting within ATLOs, marked by GAP-43 and Synapsin, forming ultrastructural synapse-like contacts within nanometer proximity to CD45^+^ immune cells (7). These neuro-immune junctions function as dynamic interfaces for rapid sensing and modulation of immune activity within the vascular wall.

Collectively, NICIs and ATLOs convert the adventitia into a neuroimmune nexus—capable of integrating, amplifying, and transmitting vascular inflammatory signals through bidirectional neuroimmune communication. These findings shift the paradigm of atherosclerosis from a purely metabolic and inflammatory disease to one with defined neuroimmune architecture and functionality. An unresolved challenge is reconciling the anti-inflammatory and vasoprotective actions of CGRP with the potentially deleterious effects of *de novo* innervation and NICI/ATLO expansion during atherogenesis, whether these represent stage-dependent phenomena or context-specific signaling outcomes remains to be determined. Therapeutic targeting of specific NICI components, such as reinforcing CGRP-mediated vascular protection, restoring vagal α7nAChR signaling, or blocking maladaptive sympathetic outputs, may offer novel strategies for halting plaque progression and reducing cardiovascular events. Future studies should prioritize the temporal dynamics, cell-specific interactions, and plasticity of NICIs across disease stages to enable precision neuromodulation in atherosclerosis.

## 6 Therapeutic opportunities targeting the neuroimmune axis in atherosclerosis

Recent advances in neuroimmunology have highlighted the therapeutic potential of modulating neural circuits to restrain vascular inflammation and atherogenesis. Traditional approaches to atherosclerosis management primarily target lipid metabolism and systemic inflammation; however, these strategies often overlook the critical contribution of neuroimmune interactions in disease progression. The identification of NICIs and neuromodulatory targets such as α7nAChRs, TRPV1 channels, and neuropeptides (e.g., PACAP, CGRP) has opened new avenues for intervention. Therapeutic strategies that restore autonomic balance, modulate neuroimmune signaling, or disrupt pathogenic nerve–immune crosstalk offer a promising adjunct or alternative to conventional treatments. This section reviews emerging modalities—including bioelectronic devices, pharmacologic agonists, and anti-cytokine therapies—with a focus on their mechanistic underpinnings, preclinical efficacy, and translational challenges in the context of atherosclerosis (**Table 1**).

**Table 1:** Comparative overview of neuroimmune-targeted therapies in atherosclerosis.

Therapeutic strategy	Representative modalities	Targets & mechanisms	Main effects	Advantages	Limitations
Bioelectronic Neuromodulation	VNS, auricular VNS	α7nAChR activation, vagal afferents, ↓ sympathetic tone	↓ TNF-α, IL-1β; ↑ M2 macrophages; ↓ plaque burden	Non-pharmacologic, adjustable, fast-acting	Low selectivity, anatomical variability, side effects
Pharmacologic Neuromodulation	α7nAChR agonists, PNU-120596	Cholinergic anti-inflammation, integrin trafficking	↓ cytokines; ↓ monocyte infiltration; ↑ phagocytosis	No implant, systemic delivery, defined targets	Desensitization, off-target α7 effects, safety unclear
Cytokine Inhibition	Canakinumab, tocilizumab	IL-1β/IL-6 blockade, TRPV1-CGRP modulation	↓ inflammation, ↓ MACE, possible sensory desensitization	Clinically validated, systemic inflammation control	Infection risk, vagal suppression, broad effects
Peptidergic/NICI Targeting	PACAP (Maxadilan), TRPV1 antag., CGRP modulators	Neuroimmune interface, adventitial nerves	↓ inflammation, ↓ macrophages, plaque protection	Lesion-specific, high precision, synergistic potential	Preclinical only, anatomical heterogeneity, peptide side effects
Combination/Future Approaches	VNS + drugs, nanoparticles, transcutaneous VNS	Multi-axis modulation: immune, neural, vascular	Synergistic effects, reduced toxicity, personalization	Precision-targeted, modular strategies	Pharmacokinetics, complexity, limited validation

This table summarizes key therapeutic strategies targeting the neuroimmune axis in atherosclerosis, including bioelectronic neuromodulation (e.g., vagus nerve stimulation), pharmacologic α7nAChR agonists, cytokine inhibitors with neuroimmune effects, peptide-based interventions targeting neuroimmune cardiovascular interfaces (NICIs), and emerging combination approaches. Each strategy is compared in terms of molecular targets, anti-inflammatory mechanisms, therapeutic benefits, and translational challenges.

Before discussing dedicated neuromodulatory strategies, it is worth noting that several current frontline atherosclerosis therapies exert incidental effects on inflammatory and autonomic pathways. For example, high-intensity statins not only lower LDL-C but also reduce vascular macrophage activation and circulating IL-6 levels, effects associated with modest improvements in heart rate variability, a surrogate of parasympathetic tone (94, 95). PCSK9 inhibitors similarly attenuate arterial wall inflammation, potentially through reduced monocyte recruitment (96). Beyond lipid-lowering agents, GLP-1 receptor agonists dampen systemic cytokine production and have been shown to decrease sympathetic nerve activity in hypertensive patients (97). Likewise, SGLT2 inhibitors can improve autonomic balance and lower plasma norepinephrine in heart failure cohorts (98). While these effects are secondary to their primary indications, they highlight that modulation of the inflammatory, autonomic axis is already occurring in clinical practice. However, emerging neuromodulatory interventions, such as vagus nerve stimulation or selective α7nAChR agonists, offer the prospect of more precise and potent targeting of pathogenic neuroimmune circuits in atherosclerosis.

### 6.1 Bioelectronic neuromodulation: vagus nerve stimulation

Preclinical studies support the efficacy of vagus nerve stimulation (VNS) as a targeted anti-inflammatory approach in atherosclerosis. While direct evidence in ApoE^⁻/⁻^ mice is limited, pharmacological activation of α7nAChRs, a key mediator of vagal signaling, significantly reduces plaque burden, promotes M2 macrophage polarization, and lowers IL-1β and TNF-α levels (99).

A recent study of a novel small-molecule agonist, AZ6983, confirmed the central role of α7nAChR: in ApoE^⁻/⁻^ mice AZ6983 reduced plaque burden by ~37–49 % versus control, dampened serum cytokines, and increased macrophage phagocytic capacity, recapitulating key functional endpoints attributed to VNS (100). Complementing these findings, imaging in human atherosclerotic vessels identified α7nAChR expression co-localized with macrophage-rich areas, lending translational significance to the mouse data (100).

Mechanistically, α7-mediated anti-inflammatory effects extend beyond cytokine suppression. A study demonstrated that macrophage recruitment into inflamed tissue depends on α7nAChR regulation of integrin αMβ2 expression; macrophages lacking α7 signal showed impaired migration in 3D matrices and *in vivo* endotoxemia models (101). These findings help explain how VNS and α7 agonism modulate plaque cellularity and stability by restricting monocyte/macrophage infiltration and favoring efferocytosis.

Despite therapeutic promise, VNS efficacy depends heavily on stimulation parameters. Low-frequency stimulation (1–10 Hz) primarily activates afferent fibers and suppresses sympathetic output, while higher frequencies (20–30 Hz) engage broader autonomic responses, including cardiac effects (102). Efforts to achieve fiber-selective targeting in VNS are challenged by significant anatomical variability and inconsistent electrode positioning in humans. The vagus nerve’s fascicular organization exhibits considerable interindividual differences, complicating reproducible and precise activation of desired fiber populations using cuff electrodes (103). Moreover, long-term clinical use of VNS can produce side effects such as bradycardia and hoarseness, which may impact patient compliance and quality of life (104); rare but serious cardiac adverse events like periodic bradycardia have also been reported, especially in pediatric cases (105). These technical and physiological limitations, combined with individual differences in disease stage and comorbidities, remain key barriers to the widespread clinical translation of fiber-selective VNS therapies.

### 6.2 Precision neuromodulatory pharmacology

α7nAChR agonists represent a pharmacologic route to harness the anti-inflammatory benefits of VNS without implantable devices. AZ6983 offers proof-of-concept: systemic administration in ApoE^⁻/⁻^ mice reduced plaque burden, suppressed TNF-α, and enhanced macrophage phagocytosis via α7 engagement (100). Translational relevance is bolstered by evidence that α7nAChR is expressed in human atherosclerotic plaques, suggesting direct targeting of lesion macrophage populations may be feasible (106).

Beyond cytokine regulation, recent *in vivo* model of endotoxemia have demonstrated a distinct role for α7nAChR in regulating immune cell trafficking: α7nAChR deficiency markedly impairs macrophage recruitment to inflamed organs, including lungs and liver, by downregulating key integrins such as αMβ2, thereby limiting integrin-mediated migration (101). These findings underscore α7 agonists’ potential not only to suppress pro-inflammatory cytokine production but also to prevent immune cell accumulation in atherosclerotic plaques via modulation of integrin-dependent macrophage trafficking.

While these features suggest a degree of pathway specificity, their actual spatial precision *in vivo* remains an open question. For example, work by Simon et al. (11), indicates that in the splenic anti-inflammatory reflex, key α7nAChR-dependent steps may occur in upstream autonomic ganglia such as the celiac ganglion, rather than solely on plaque-resident macrophages. This raises the possibility that systemic α7 agonists might influence multiple anatomical sites along the reflex arc, not all of which are disease specific.

These pharmacologic strategies offer precision over systemic anti-inflammatories. Targeting α7nAChR allows pathway-specific modulation of immune responses, minimizing off-target immunosuppression (107). Moreover, allosteric modulators like PNU-120596 significantly enhance receptor responsiveness, enabling lower dosing and increased specificity in reducing inflammatory signaling (108).

However, concerns remain: α7 agonists must avoid desensitization of receptor function and do not yet have established long-term safety profiles in vascular patients (109). Another unresolved issue is tissue specificity, α7nAChR is expressed in multiple cell types (e.g. neurons, epithelial cells), raising potential for unintended effects unless cell-targeted delivery approaches are employed (110).

Efforts are ongoing to develop next-generation α7 modulators with biased signaling properties or restricted biodistribution. Combining pharmacologic agents with localized VNS or transcutaneous auricular VNS may synergize inflammatory suppression while reducing systemic exposure. Overall, precision α7 pharmacology offers a promising, device-independent alternative to neuromodulation, but its optimal targeting strategy in atherosclerosis will require further refinement and experimental validation.

### 6.3 Anti-inflammatory drugs with neuroimmune effects

Study highlights that beyond conventional lipid-lowering therapies, anti-cytokine agents also exert profound modulatory effects on neuroimmune circuits involved in atherosclerosis. The landmark CANTOS trial demonstrated that inhibiting IL-1β with canakinumab reduces major adverse cardiovascular events without altering lipid profiles, supporting the immunomodulatory relevance of IL-1β signaling in plaques (4). While CANTOS itself did not directly assess neural endpoints, more recent preclinical studies indicate that IL-1β can also modulate arterial nociceptive sensory fibers and neuroimmune signaling (6), thereby providing a mechanistic link between cytokine blockade and potential effects on vascular innervation. Genetic deletion of IL-1β in ApoE^−^/^−^ mice leads to a significant reduction in plaque burden, accompanied by decreased monocyte adhesion and inflammatory cytokine expression (111). Intriguingly, IL-1β has been shown to sensitize TRPV1^+^ sensory neurons in inflammatory skin and joint diseases, promoting neuropeptide release and local immune amplification (112). Given the established role of TRPV1–CGRP signaling within NICIs in atherosclerosis, it is plausible that IL-1β blockade might also dampen neurogenic inflammation in vascular lesions. While direct evidence for IL-1β–TRPV1 crosstalk in atherosclerosis is lacking, future investigations could reveal whether sensory neuroimmune desensitization contributes to the plaque-stabilizing effects of anti-cytokine therapy.

IL-6 receptor antagonism, using agents like tocilizumab, may provide benefits that extend beyond standard anti-inflammatory effects. Although direct evidence in ApoE^⁻/⁻^ mice linking IL-6R blockade to modulation of plaque-associated sympathetic fibers remains unavailable, preclinical research indicates that IL-6 signaling robustly influences sympathetic nervous system activity. For example, intracerebroventricular IL-6 administration in rats was found to significantly increase splenic sympathetic nerve discharge and elevate circulating norepinephrine levels, highlighting a central IL-6–driven sympathetoneural axis (113). Additionally, systemic IL-6 elevation is commonly associated with increased sympathetic tone in chronic inflammatory conditions, providing a mechanistic link to neuroimmune modulation in vascular disease (113, 114). These findings support a plausible dual mechanism: IL-6R blockade could suppress inflammatory signaling and indirectly attenuate sympathetic-driven neuroimmune activation in atherosclerosis. However, targeted *in vivo* studies are needed to confirm whether IL-6R-targeted therapies modulate sympathetic innervation or β_2_-adrenoceptor expression in plaque-associated nerves.

These immunomodulators differ from traditional neuromodulatory therapies. Whereas VNS and pharmacologic agonists directly target neural circuits, cytokine inhibitors reprogram neuroimmune interactions indirectly, quieting both inflammatory triggers and sensitized neural feedback loops. While no studies have directly examined the combined effects of VNS and canakinumab in LDLR^⁻/⁻^ mice, their complementary mechanisms of neural circuit modulation and cytokine inhibition suggest the potential for synergistic benefits in reducing plaque size and systemic inflammation. Future experimental work is needed to evaluate this possibility.

However, targeting cytokines is not without challenges. Systemic neutralization of IL-1β or IL-6 carries infection risk, as these mediators are essential for host defense. For instance, the CANTOS trial reported significantly elevated rates of serious infections and sepsis among individuals receiving canakinumab compared to placebo (4). Moreover, IL-1β antagonists can blunt fever-induced vagal reflexes, potentially shifting autonomic balance—animal studies show that subdiaphragmatic vagotomy blocks IL-1β–induced fever and behavioral changes, underscoring the role of vagal afferents in mediating inflammatory responses (115). Emerging clinical data also link sustained IL-6 suppression to transient vagal hypoactivity: heart rate variability (HRV), a key measure of parasympathetic function, inversely correlates with inflammatory markers such as IL-6 and CRP in patients with rheumatoid arthritis (116). These observations underscore the need for therapies that selectively modulate pathogenic neuroimmune pathways while preserving beneficial autonomic reflexes.

In summary, anti-cytokine agents have proven efficacy in cardiovascular trials and show emerging neuromodulatory effects by quelling both inflammatory and neural feedback. However, their non-specific nature raises safety concerns. Combinatorial approaches with electrical or pharmacologic neuromodulation may achieve greater precision and reduced side effects, which sheds light on a balanced “neuro-cytokine” therapeutic axis for atherosclerosis.

### 6.4 Future directions: targeting key neuroimmune pathways

The evolving understanding of plaque-centric neuroimmune systems, NICIs, creates fertile ground for novel therapies that directly target nerve–immune interfaces within the adventitia. For instance, Sensory TRPV1 signaling has been implicated in modulating vascular–sympathetic reflexes and homeostatic blood pressure regulation, suggesting that TRPV1 antagonists *may* offer a way to interrupt stress-induced pro-inflammatory circuits—though direct evidence in atherosclerotic plaque models remains pending (117). Separately, centrally administered gabapentin modulates blood pressure and heart rate via the NTS in hypertensive rats through NOS-dependent mechanisms, indicating its capacity to recalibrate autonomic reflexes—though synergy with α7nAChR agonists in AS has yet to be explored (118).

Within peptide neuromodulators, PACAP and CGRP have emerged as promising yet divergent targets. As mentioned, PACAP–PAC1 activation via Maxadilan inhibits macrophage TNF-α production and increases regulatory IL-10 expression in murine plaques independent of lipids (76). Conversely, CGRP antagonism, widely used in migraine, presents a cautionary tale: galcanezumab-treated ApoE^⁻/⁻^ mice exhibit increased plaque macrophage infiltration and vessel wall inflammation, highlighting CGRP’s protective role in vascular homeostasis (119). These findings have major translational implications—identifying CGRP inhibitors as potentially contraindicated in high-risk cardiovascular patients, while spotlighting PACAP agonists as therapeutic candidates.

Challenges loom in targeting NICIs and peptidergic systems. Anatomical heterogeneity across arterial beds may limit drug access, and long-term manipulation of sensory fibers may disrupt homeostatic neurovascular reflexes, as sensory innervation density and function vary widely by vascular territory (120). Moreover, biased agonism at PAC1 receptors may differentially modulate vascular versus neural signaling, with PAC1-mediated vascular relaxation and central neural sensitization showing distinct pharmacology—necessitating careful pharmacodynamic profiling (121).

However, the emerging clinical use of CGRP blockade in migraine and the translational potential of PAC1 agonists such as Maxadilan underscore the urgent need to evaluate cardiovascular safety and long-term immunomodulatory outcomes in relevant patient populations. Future studies should systematically map neuropeptide expression dynamics and receptor distribution across atherosclerosis stages to inform the rational design of targeted interventions.

Nevertheless, integrating structural NICI mapping with focused neuromodulation holds promise. Combined approaches, e.g., nanoparticle-mediated PACAP delivery to adventitial nerve sheaths, or localized TRPV1 blockade using targeted antibodies, could allow lesion-specific intervention, although agonist such as capsaicin should be avoided due to its side effects (122, 123). These strategies may showcase integrated mechanistic insight and therapeutic innovation. Overall, therapy aimed at disrupting pathological nerve–immune crosstalk, via PACAP agonists, CGRP modulators, and NICI-directed interventions, offers a new frontier in precision atherosclerosis management.

### 6.5 Challenges in clinical translation

Several translational barriers complicate the therapeutic potential of targeting neuroimmune pathways in AS application in human disease. One major obstacle lies in the anatomical and functional complexity of autonomic circuits. VNS, while potently anti-inflammatory in ApoE^⁻/⁻^ mouse models via activation of α7nAChR, expressing macrophages, often results in non-specific activation of adjacent neural branches when translated into larger animals or humans, due to the anatomical complexity and lack of fiber-type selectivity with conventional cuff electrodes (124). Standard cuff electrodes lack fiber-specific resolution, inadvertently activating laryngeal, leading to coughing or bradycardia (124, 125). Even in recent animal studies (adult male Sprague-Dawley rats and Merino ewes) employing closed-loop or optogenetic approaches, achieving selectivity for anti-inflammatory vagal efferents remains technically demanding and largely untested in AS-specific contexts (126).

Beyond technical hurdles, the systemic effects of neuromodulatory peptides, such as CGRP and PACAP, raise concerns about long-term safety and vascular specificity. CGRP receptor antagonists (e.g., erenumab) are now widely used for migraine prophylaxis, and large-scale post-marketing data suggest they do not increase major adverse cardiovascular events (127). However, these findings are based on low-risk populations and may not fully translate to individuals with established atherosclerosis, where CGRP plays a vasoprotective and anti-inflammatory role. Indeed, ApoE/CGRP double-knockout mice develop significantly larger plaques and enhanced macrophage activation, underscoring α-CGRP’s protective role in atherogenesis (119). Conversely, PACAP-based therapies remain at the preclinical stage: the PAC1 receptor agonist Maxadilan markedly reduced plaque incidence (from ~75 % to ~28 %) and lumen stenosis in ApoE^^−^/^−^^ mice, lowered TNF-α^+^ and IL-1β^+^ areas, and decreased apoptosis in lesions—even under cholesterol-enriched diets (76). However, these effects are confined to murine models; no safety or efficacy data exist in non-rodent species, and translation to humans remains speculative.

Another challenge is the striking inter-individual variability in autonomic tone, inflammatory responses, and neural-immune coupling across patients. Factors such as age, metabolic syndrome, autonomic neuropathy, and even sleep quality alter neuroimmune balance, potentially modulating response to interventions. For instance, HRV, widely used as a proxy for vagal tone, is inversely associated with circulating markers of inflammation in cardiovascular disease cohorts: lower HRV correlates with elevated IL-6, CRP, and fibrinogen levels (128–130). Additionally, in a large young adult cohort (N = 2,064), reduced HRV was independently associated with higher monocyte and leukocyte counts, further suggesting HRV as a physiological biomarker of immune activation (131). These findings underscore the need for physiological stratification using HRV or related autonomic markers to identify individuals most likely to benefit from neuromodulatory therapies.

Adding further complexity, recent evidence indicates that VNS does not always act through the canonical α7nAChR pathway. In inflammatory disease models of acute lung injury, VNS-induced immunosuppression was abolished by adrenalectomy, but not by α7nAChR blockade, suggesting a vagus–adrenal–catecholamine axis rather than direct cholinergic signaling (132, 133). This distinction is crucial for AS, where peripheral nerve terminals may act via local versus systemic pathways, making target engagement and mechanistic verification essential in future trials.

Finally, chronic neuromodulation may induce neural plasticity or receptor desensitization, as seen in prolonged CGRP or PACAP exposure, potentially diminishing efficacy over time (134). Despite their promising immunomodulatory profiles, long-term studies evaluating tolerance, feedback inhibition, or compensatory sympathetic activation are largely absent in current AS models. Moreover, while bioelectronic and pharmacologic therapies hold distinct promise, their combined effects, whether synergistic or antagonistic, remain unexplored, raising critical questions for therapeutic design.

In conclusion, advancing neuroimmune-targeted therapies for atherosclerosis will require refined neuromodulation tools with enhanced circuit selectivity, rigorous safety validation of peptidergic drugs, and patient stratification approaches grounded in real-time autonomic and inflammatory biomarkers. Addressing these challenges through integrated engineering, pharmacological, and immunological frameworks is essential to realize the clinical potential of this emerging field.

## 7 Conclusion

Mounting evidence positions neuroimmune dysregulation as a central mechanism linking autonomic imbalance to chronic vascular inflammation and atherogenesis. Disruptions in sympathetic-parasympathetic tone, aberrant neuropeptide signaling, and the pathological remodeling of NICIs collectively shape the inflammatory milieu of the arterial wall. These neurogenic cues not only influence local immune cell activation and trafficking but also modulate hematopoietic niches and systemic cytokine output, reinforcing a feed-forward loop of vascular injury. Preclinical studies have highlighted the therapeutic potential of neuromodulatory interventions, including selective α7nAChR agonists, VNS, and neuropeptide-targeting strategies, to attenuate immune activation and stabilize atherosclerotic plaques. However, significant translational hurdles remain, particularly in achieving spatial precision, minimizing off-target autonomic effects, and tailoring therapies to interindividual variability in neural-immune architecture. Moving forward, integrating neuroimmunology with systems-level vascular biology holds promise for the rational design of precision therapies that transcend lipid-lowering paradigms. Such strategies may ultimately reshape the clinical approach to atherosclerosis, offering durable immunomodulation and improved cardiovascular outcomes.
